# Antibiotic Therapy in Integrated Oncology and Palliative Cancer Care: An Observational Study

**DOI:** 10.3390/cancers14071602

**Published:** 2022-03-22

**Authors:** Martine Kjølberg Moen, Erik Torbjørn Løhre, Gunnhild Jakobsen, Morten Thronæs, Pål Klepstad

**Affiliations:** 1 Clinic of Anaesthesia and Intensive Care, St. Olav’s University Hospital, 7030 Trondheim, Norway; pal.klepstad@ntnu.no; 2 Cancer Clinic, St. Olav’s University Hospital, 7030 Trondheim, Norway; erik.t.lohre@ntnu.no (E.T.L.); gunnhild.jakobsen@ntnu.no (G.J.); morten.thrones@ntnu.no (M.T.); 3 Department of Clinical and Molecular Medicine and Health Sciences, NTNU—Norwegian University of Science and Technology, 7030 Trondheim, Norway; 4 Department of Public Health and Nursing, Faculty of Medicine and Health Sciences, NTNU—Norwegian University of Science and Technology, 7030 Trondheim, Norway; 5 Department of Circulation and Medical Imaging, Faculty of Medicine and Health Sciences, NTNU—Norwegian University of Science and Technology, 7030 Trondheim, Norway

**Keywords:** palliative cancer care, integrated oncology and palliative care, detecting infections, antibiotic treatment, symptom development, survival

## Abstract

**Simple Summary:**

Approximately one-quarter of the patients with advanced cancer acutely admitted to the Palliative Care Unit at St. Olav’s University Hospital received intravenous antibiotics. We observed that physiological variables and paraclinical findings in patients with and without infections differed at admission but observed no differences in patient-reported outcome measures. Patients admitted for infection had no shorter life expectancy than patients without infections. We did not observe any difference in the prescription of antibiotics to patients with ongoing anti-cancer therapy (integrated pathway) compared to patients with no ongoing cancer therapy (palliative care pathway). This information increases the knowledge about the use of antibiotic therapy in palliative cancer care.

**Abstract:**

Decision-making for antibiotic therapy in palliative cancer care implies avoiding futile interventions and to identify patients who benefit from treatment. We evaluated patient-reported outcome-measures (PROMs), physiological findings, and survival in palliative cancer care patients hospitalized with an infection. All acute admissions during one year, directly to a University Hospital unit that provided integrated services, were included. Serious infection was defined as a need to start intravenous antibiotics. PROMs, clinical and paraclinical variables, and survival were obtained. Sixty-two of 257 patients received intravenous antibiotic treatment. PROMs were generally similar in the infection group and the non-infection group, both in respect to intensities at admission and improvements during the stay. There were more physiological and paraclinical deviations at admission in patients in the infection group. These deviations improved during the stay. Survival was not poorer in the infection group compared to the non-infection group. Patients in integrated cancer care were as likely to be put on intravenous antibiotics but had longer survival. In integrated oncology and palliative cancer services, patients with an infection had similar outcomes as those without an infection. This argues that the use of intravenous antibiotics is appropriate in many patients admitted to palliative care.

## 1. Introduction

Early access to palliative care services in oncology is advocated [1]. In addition, novel treatment options have resulted in extended survival for more cancer patients [2]. By its original definition, palliative care intends to neither hasten nor postpone death [3]. However, for patients receiving both oncological treatment and palliative care, both life prolongation and symptom relief are valid treatment goals and therefore antibiotic treatment can be indicated [4,5]. On the other hand, a systematic review reported non-beneficial use of antibiotics in more than a fifth of dying patients [6]. Both international and national guidelines refrain from recommending antimicrobial therapy for symptom management in end-of-life care [7,8]. These recommendations are based on the assumption that the patients are facing imminent death, and do not necessarily include considerations on the incidence of severe infections in palliative care patients with longer expected survival, or how these infections affect symptom burden and survival in this group of patients [4].

Patients with advanced cancer are prone to infections due to cancer-treatment-induced immunosuppression, barrier dysfunctions, and the use of immunomodulating drugs like corticosteroids [9]. Antibiotic treatment in palliative care patients may not necessarily aim for, or result in, life prolongation, albeit some studies demonstrated an improved survival after the successful treatment of infections [10,11]. However, a literature review found no clear association between the use of antibiotics and survival in palliative care patients [4]. Included in the review, was an older study demonstrating symptom relief for urinary and respiratory tract infections treated with antibiotics in palliative care cancer patients [12]. Another prospective study also reported improvement in dysuria and cough with antibiotic treatment in palliative care patients, even though one-quarter of the patients died within one week of antibiotic administration [13]. Most studies are based on a retrospective design, and a systematic review on the role of antimicrobial therapy for symptom management in palliative care patients concluded that data to guide decision-making is limited [14]. The review also stated that future studies on the topic should systematically measure symptom responses [14]. Identification and treatment of physical symptoms in palliative care patients are important to prevent and relieve suffering [15]. When considering antibiotic therapy in palliative care, the potential benefits must outweigh the side effects of antimicrobial treatment. For the individual, the use of antibiotics may cause drug interactions, allergic reactions, antibiotic-associated diarrhea and colitis, and hence more hospital admissions [16]. For society, liberal use of antibiotics may contribute to the emergence of more antibiotic-resistant bacteria and higher costs [17]. In palliative care patients, these potential challenges must be weighed against the antibiotics’ potential for symptom relief and life extension [4].

To provide useful treatment and to avoid futile interventions are important principles in palliative care and in medicine in general [18]. Selecting which patients will benefit from antibiotic treatment in a patient population with clinical and paraclinical features resembling patients with infectious diseases, adds further complexity. A growing focus on the early integration of palliative care into oncology makes this selection process even more important [1]. Thus, studies on implications of antibiotic treatment in palliative cancer care are warranted both to limit over- and undertreatment. We report the use of antibiotic therapy, patient-reported outcome measures (PROMs), paraclinical and clinical findings, and survival in palliative care cancer patients with clinically evident infections admitted to an acute palliative care unit (APCU). With the aim to investigate differences and similarities, we compared patients with infection-related admissions and non-infection related admissions and patients who received integrated oncology services and standard palliative cancer care.

The following research questions were addressed:

1. What are the differences and similarities in PROMs and clinical and paraclinical features at admission for palliative care cancer patients with and without infections?
2. What are the differences and similarities in the development of symptoms and clinical and paraclinical features during the hospital stay for palliative cancer patients with and without infections?
3. Are acutely admitted patients receiving integrated oncology services more often treated with intravenous antibiotics compared to patients receiving palliative cancer care only?
4. What is the survival for patients with advanced cancer, receiving integrated oncology and palliative care services, and treated for infections necessitating acute hospital admission?

## 2. Materials and Methods

### 2.1. Study Design

An observational study was conducted at the APCU, Cancer Clinic, St. Olav’s hospital, a 1000-bed university hospital located in Trondheim, Norway. The APCU has 12 beds and approximately 450 admissions a year. Adult (≥18 years) cancer patients with incurable disease admitted between 15 January 2019 and 15 January 2020 were included. Patients with hematological, gynecological, and pulmonary cancer are admitted to the APCU only when in need of neuraxial pain management. APCU’s inpatient programs facilitate early integration of oncology and palliative care and provide aggressive symptom management. Patients are treated by a multidisciplinary team of senior consultants with specialized training in palliative care, oncologists responsible for tumor-directed treatment, nurses, physiotherapists, social workers, and clinical dietitians.

The current paper is a retrospective secondary analysis of a previously published study on the delivery of palliative care [19]. In the present analysis, we identified all acute hospitalizations of patients with a clinically-based diagnosis of infection who received intravenous antibiotics at admission. This group of patients was compared to all patients acutely admitted due to other reasons, the non-infection group. Patients with ongoing antibiotic treatment at admission, patients initiated on oral antibiotics at admission, and patients admitted for other reasons but diagnosed with infections later during the hospital stay were excluded from the analysis. Palliative patients with ongoing oncological treatment were defined as receiving integrated oncological services. Cancer patients in whom the systemic tumor-directed therapy (other than castration for prostate cancer) was withdrawn were classified as receiving palliative care only. Follow-up of patients in integrated cancer care is shared between the treating oncologist and the palliative care team, while patients in palliative cancer care solely were treated by the palliative care team.

### 2.2. Data Collection and Assessments

The design of the data collection is described elsewhere [19]. The patients reported symptom scores on the 11-point numeric rating scale (NRS 0-10) [20]. Assessments on average pain and worst pain, tiredness, drowsiness, nausea, reduced appetite, shortness of breath, depression, anxiety, well-being, sleep and constipation were registered at admission and at discharge but no later than day 10 to ensure sufficient time to achieve antibiotic treatment responses and to reduce the risk of registering changes due to reasons other than the initial infection [21,22]. Oncologists with palliative care expertise reported cancer diagnosis, metastatic status, Eastern Cooperative Oncology Group (ECOG) [23] performance status, medical comorbidity, and interventions during the hospital stay. Infections were diagnosed and treated by the attending physicians. The infection was considered as serious if in need of intravenous antibiotics. Reviews of the medical records identified antibiotic treatment and duration, suspected sources of infection, physiological variables as assessed by the national early warning score (NEWS) [24], clinical chemistry tests (C-reactive protein (CRP), leucocytes and neutrophiles), blood and other cultures, hospital length of stay (LOS), and survival [19].

### 2.3. Statistical Analysis

Descriptive statistics were used, with means or medians as descriptors of central tendencies, and a range as descriptors of dispersion; with a 95% confidence interval (CI) for survival. The Chi-Square test was used to evaluate the association between the occurrence of infection or antibiotic use in integrated cancer care and palliative cancer care. Group comparison at admission for the infection group and the non-infection group was analyzed by independent sample *t*-test. For the development of symptom intensities, clinical and paraclinical parameters were performed through a calculation of difference in observations from admission to discharge (but no later than day 10 of the hospital stay), and the infection group and non-infection group were compared by use of an independent *t*-test. Mean changes in symptom scores from admission to discharge (or day 10) were reported for patients both in the infection group and non-infection group and evaluated by using the two-tailed paired sample *t*-test. A two-sided *p*-value ≤ 0.05 was considered statistically significant. No sample size calculations were performed.

### 2.4. Ethics

The Regional Committee for Medical Research Ethics, Health Region Central Norway (REK) (2018/925/REK Midt) defined the project as healthcare improvement, without the need for explicit informed consent from the patients. The journal review was approved by the Regional Committee for Medical Research Ethics, Health Region South-East (2020/107564).

## 3. Results

### 3.1. Inclusions and Exclusions

**Figure 1:**
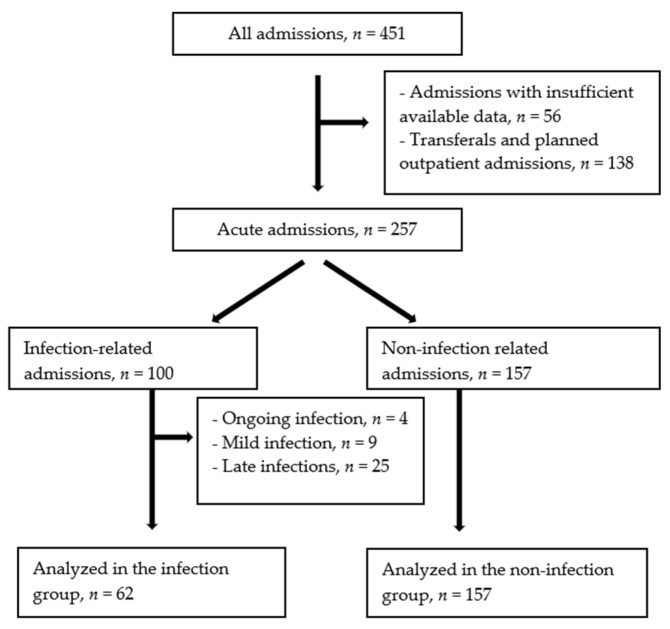
Included and excluded admissions during the one year study period.

During the one year study period, 451 admissions were registered at the APCU. Out of these, 100 acute admissions were classified as infection-related. In 38 admissions, the patients were either already on antibiotics (ongoing infection), per oral antibiotics that were started at admission (mild infection), or the infection was diagnosed later than 24 h after hospitalization (late infection). These admissions were excluded from the analysis. The remaining 62 admissions were considered serious infections and intravenous antibiotic treatment was initiated at admission, which constituted the infection group. The comparison group comprised 157 acute admissions due to reasons other than infections, henceforth referred to as the non-infection group (Figure 1).

### 3.2. Patient Demographics

For all patients, the mean patient age was 69 years (range 29–98) and 60% were males. The most common primary diagnoses were gastrointestinal (52%), urological (23%), and breast cancer (9%). Eighty-eight percent of the patients had metastatic disease, more than half of the patients had ECOG performance status III or IV, and close to 40% of the patients received integrated oncological services. Patient demographics for the infection group and the non-infection group are shown in Table 1. The mean age for patients in the infection group was 66 years and 69% were males. Thirty-nine percent of the admissions in the infection group belonged to integrated cancer care versus 36% in the non-infection group. The mean hospital LOS was 7.0 days.

### 3.3. Infection Characteristics

Pneumonia was the most frequently suspected primary source of infection, followed by urinary tract and gastrointestinal infections (Table 2). The source of infection was unknown for almost one-third of the patients and approximately one-tenth had multiple sites of infection. Blood cultures were drawn from 54 out of the 62 admissions in the infection group (87%). Seventy-seven percent of the blood cultures were negative. Correspondingly, urine cultures were obtained from 44 out of 62 patients (71%). Fifty-two percent of the urine cultures were negative. Further details are provided in Table 2, which also delineates the use of antibiotics.

### 3.4. Group Comparison at Admission

Details on the differences and similarities in symptoms and clinical and paraclinical features for patients admitted with and without serious infections are described in Table 3. At admission, NRS sleep scores were significantly higher in the infection group than in the non-infection group (5.0 vs. 3.9, respectively, *p* = 0.02). There were no other significant differences in PROM scores between the groups. Furthermore, heart rate, temperature, total NEWS score, and CRP were all significantly higher in the infection group compared to the non-infection group.

### 3.5. Dynamic Group Comparison during the Hospital Stay

The development in PROMs and clinical and paraclinical findings during the hospital stay for patients admitted with and without serious infections are displayed in Table A1 and Table A2. There were no significant group differences with regard to PROMs during the hospital stay. For infection patients, there was a significant increase in systolic blood pressure, and significant decreases in heart rate, temperature, total NEWS score, and CRP during the hospital stay compared to the non-infection group (Table 4). Leukocyte and neutrophile counts decreased during hospitalization in the infection group (*p* = 0.06 and *p* = 0.008, Table A1), no change was noted in the non-infection group (Table A2).

### 3.6. Antibiotic Treatment and Survival in Patients with and without Integrated Oncology Care

Of the acutely admitted patients receiving integrated oncology care, 30% were treated with intravenous antibiotics at admission. The corresponding percentage of patients receiving palliative care was only 27% (Table A3). Radiological examinations and other interventions were similar in the integrated cancer care and the palliative cancer care group except for MRI examinations and surgery, which were more prevalent in the integrated cancer care group (Table A3). Patients with integrated cancer care have longer survival rates than patients receiving palliative cancer care only; median days (95% CI) 94 (55–113) versus 28 (23–37). In acutely admitted patients with an infection receiving integrated oncology services, median (95% CI) survival after hospitalization was 108 days (69–166). The corresponding number for patients with infection receiving palliative care was only 41 days (25–59).

## 4. Discussion

### 4.1. Statement of Principal Findings

We observed that approximately one-quarter of the patients acutely admitted to the APCU were diagnosed to have a serious infection. Patients with and without infections were relatively similar in terms of PROMs at admission. Clinical and paraclinical parameters typical for patients with serious infections differed at admission in patients in the infection group and the non-infection group. There were no group differences in symptom development during the hospital stay. Increments in systolic blood pressure and decrements in heart rate, temperature, total NEWS score, and CRP were typical occurrences during the hospital stay for patients acutely admitted with serious infections. Patients who received integrated oncological services were not more prone to be admitted with serious infections. However, the life expectancy of patients receiving integrated oncological services and acutely admitted with serious infections was much longer than for the corresponding patients receiving palliative care only.

### 4.2. Appraisal of Methods

The current paper presents a secondary analysis of a study originally designed to evaluate interventions and symptom relief for patients with incurable cancer admitted to an APCU providing integrated oncology and palliative care services. [25]. The PROMs were obtained prospectively, as planned for the primary study, while the physiological observations, clinical chemistry results, use of antibiotics and culture results were documented as standard care. Thus, we had no standardized study procedure for the diagnosis and sampling with respect to infection detection [26]. Nevertheless, clinical practice was carried out by a small team of experienced palliative care physicians and therefore should be consistent across the patient sample. An inherent limitation in studies on cancer patients and infections is the fact that cancer can cause elevated levels of pro-inflammatory markers and clinical findings, thus mimicking an ongoing infection [27]. Finally, this being an explorative study, we did not adjust for multiple testing.

### 4.3. Comparison with Previous Work

Studies describing the use of antibiotics therapy in palliative care are mainly based on data collected before the introduction of integrated oncology and palliative cancer care [14,28]. For decades, palliative care focused on end-of-life care [1]. In the millennium, the focus of palliative care also changed [3,29]. As exemplified in the updated WHO definition, palliative care is applicable early in the course of illness. Therefore, studies focusing on contemporary clinical reality are warranted. Despite the methodological shortcomings, our results narrate antibiotic therapy in the era of integrated oncology and palliative cancer care.

Almost ten years ago, a systematic review on antibiotics for symptom management in palliative care reported the prevalence of antimicrobial use in cancer patients ranging from 19% to 84% [14]. A later overview of antibiotic therapy in palliative care found that respiratory and urinary tract infections were prevalent and that broad-spectrum antibiotics were often applied [4]. The negative consequences of the overuse of antibiotics with a broad antimicrobial spectrum are vividly described in a recent paper on infections in palliative care [30]. Our findings support that serious infections are prevalent in palliative cancer care and that the lungs and urinary tract are common sites of infection. In addition, we observed that more than 40% of the patients were treated with penicillin or a beta-lactam/aminoglycoside combination, and in two-thirds of the patients, no change in antibiotic regimen was required.

Advanced cancer is often accompanied by increased inflammation [31]. Consequently, patients with locally advanced or metastatic cancer may exhibit clinical and paraclinical properties, indicating an ongoing infection. Fever is a traditional hallmark of infection [32]. Even though we detected significant temperature group differences at admission, for the individual advanced cancer patient with a potential infection, a slight temperature elevation may not be decisive for the diagnostic work-up. In our study, the finding of elevated values of heart rate, total NEWS score, and CRP supported the infection diagnosis. On the contrary, in our study, PROMs, and leukocyte and neutrophil counts were not much different in acutely admitted palliative care cancer patients considered being with and without infections.

In the management of patients with potentially life-threatening infections, a rapid diagnostic work-up, followed by an immediate administration of adequate antibiotics treatment, is mandatory [33]. This implies instituting antibiotics prior to available culture results. The low percentage of positive blood cultures in the current study is opposed to previous research and may possibly indicate some degree of overestimation of infections [34]. Nevertheless, our findings support current knowledge on serious infections and underline that increased systolic blood pressure and decreased heart rate, total NEWS score, and CRP during hospitalization may indicate infection control [33]. Interestingly, the development during the hospital stays in standardized patient-reported symptoms was not different for the two compared groups.

Cancer patients receiving oncological treatment are susceptible to infections [35]. In addition to neutropenic fever and sepsis, they are also prone to more serious outcomes from other infections, such as pneumonia and urinary tract infections [36]. These facts underline that the rapid initiation of antibiotics is often indicated in this group of patients. Still, we found no group differences in the use of intravenous antibiotics in patients receiving integrated oncological services and palliative care only. The study design allows no further investigations on this topic, but this finding might represent examples of both over- and undertreatment, and futile interventions [6,18].

Survival in palliative cancer care patients varies widely [37]. When palliative care is introduced early in the disease trajectory, more patients will have a substantial life expectancy. Increasing life span without reducing the quality of life is a goal of oncological treatment [38]. Modern palliative care may also contribute to reaching this goal, as described in studies of integrated oncology and palliative cancer care [1,28]. Our survival findings underline the obvious fact that oncological patients receiving early palliative care have a longer life expectancy than patients receiving palliative care later in the disease trajectory. Hence, detecting serious infections in patients receiving early integrated oncology services must be a part of modern palliative medicine.

### 4.4. Implications and Further Work

Triaging patients suitable for more intensive monitoring and care is an established principle in medicine. Our results indicate also, that in modern palliative care, the identification, and active treatment of patients with a potential for significant life prolongation are important. Due to the inherent methodological weakness of the current study, the findings should be confirmed in other studies [39]. In addition, future research should address improved measures for identifying patients with a potential for meaningful responses to antibiotic therapy in palliative cancer care.

## 5. Conclusions

Approximately one-quarter of the patients acutely admitted to an APCU had a serious infection. Patients with and without infections were relatively similar in terms of PROMs at admission and had similar improvements during the APCU stay. Survival was not poorer in patients treated for an infection. The findings suggest that intravenous antibiotic therapy is appropriate for many patients admitted to palliative care.

## Figures and Tables

**Table 1:** Demographic and baseline clinical characteristics at admission.

Characteristic		Infection Group (*n* = 62)	Non-Infection Group (*n* =157)
	Age, mean years (range)	65.8 (29–94)	70.2 (30–98)
	Gender, % male	69	56
Cancer type, *n* (%)	Gastrointestinal (GI)	27 (44)	86 (55)
	Urological	23 (37)	26 (17)
	Breast	3 (5)	17 (11)
	Lung	0	1 (1)
	Head-neck	2 (3)	13 (8)
	Other	7 (11)	14 (8)
Metastasis, *n* (%)	Yes	55 (89)	113 (72)
	No	7 (11)	14 (9)
Comorbidities, *n* (%)	Cardiovascular disease	21 (34)	22 (14)
	Diabetes	6 (10)	20 (13)
	Kidney disease	0	4 (3)
	Musculoskeletal disease	5 (8)	17 (11)
	Psychiatric disorder	3 (5)	22 (14)
	COPD ^1^	4 (7)	7 (5)
	Liver disease	1 (2)	0
	Other	14 (22)	31 (19)
ECOG ^2^ performance status score, *n* (%)	0	0	2 (1)
	1–2	26 (42)	63 (40)
	3–4	35 (57)	91 (58)
	Missing	1 (2)	1 (1)
Trajectory, *n* (%)	Palliative cancer care	37 (60)	99 (63)
	Integrated cancer care	24 (39)	57(36)
	Information not available	1 (2)	1 (1)
Radiology, *n* (%)	X-ray	37 (60)	41 (26)
	Computer tomography	28 (45)	55 (35)
	Ultrasound	7 (11)	15 (10)
	Magnetic resonance imaging	4 (7)	27 (17)
	No	10 (16)	57 (36)
Interventions *n* (%)	Radiological intervention ^3^	5 (8)	13 (8)
	Rehydration	43 (69)	65 (41)
	Nutrition (iv or nasogastric feeding tube)	10 (16)	15 (10)
	Transfusion	20 (32)	15 (10)
	Surgery	1(2)	3 (2)
	Use of multidisiplinary team	24 (39)	52 (33)
Mean hospital length of stay, mean days (range)		8.2 (2–29)	6.8 (0–39)
Survival after admission, median days (95% CI ^4^)		58.5 (41–86)	36 (28–44)
In-hospital mortality *n* (%)		8 (12.9)	22 (14.0)

^1^ Chronic obstructive pulmonary disease. ^2^ Eastern Cooperative Oncology Group. ^3^ Drain insertions and stents. ^4^ CI, confidence interval.

**Table 2:** Infections and antibiotic treatment characteristics. Infection group, *n* = 62.

Characteristic		Infection Group, *n* = 62
Source of infection, *n* (%)	Lungs	32 (52)
	Urinary	10 (16)
	GI	6 (10)
	Skin or soft tissue	1 (2)
	Other (se under)	1 (2)
	Unknown	20 (32)
	More than one focus	8 (13)
Blood culture, *n* (%)	Positive	6 (10)
	Gram-negative	4 (7)
	Gram-positive	1 (2)
	Poly-microbial or fungal BSI	1 (2)
	Negative	48 (77)
	Not taken	8 (13)
Urine culture, *n* (%)	Positive	12 (20)
	Negative	32 (52)
	Not taken	18 (29)
Other cultures, *n* (%)	Positive	2 (3)
	Negative	1 (1)
	Not taken	59 (95)
Antibiotics	Monotherapy penicillin/ampicillin	13 (21)
	2. Beta-lactam + aminoglycoside	13 (21)
	3. Second or third generation cephalosporins	13 (21)
	4. Piperacillin-tazobactam	9 (15)
	5. Carbapenems	3 (5)
	6. Group 1, 2 or 3 + metronidazole	9 (15)
	7. Quinolones: ciprofloxacin	1 (2)
	8. Other	1 (2)
Antibiotic change, *n* (%)	No	41 (66)
	Yes	21 (34)
	Due to resistance	6 (10)
	Therapy failure	7 (11)
	De-escalation	1 (2)
	Uncertain	6 (10)
Antibiotic appropriateness after resistance, *n* (%)	Yes	14 (22)
	No	4 (7)
	Negative blood culture	38 (61)
	No blood culture	6 (10)
Antibiotics after change	Monotherapy penicillin/ampicillin	2 (3)
	2. Beta-lactam + aminoglycoside	1 (2)
	3. Second or third generation cephalosporins:	4 (7)
	4. Piperacillin-tazobactam:	3(5)
	5. Carbapenems	2 (3)
	6. Penicillinase-resistant penicillins:	2 (3)
	7. Group 1, 2 or 3 + metronidazole	4 (7)
	8. Vankomycin + linezolid	1 (2)
	9. Other	2 (3)
Days of intravenous treatment, mean (range)		7.15 (1–20)

**Table 3:** Group comparison at admission.

		Infection Group, *n* = 62. Mean	Non-Infection Group, *n* = 157. Mean	*p*-Value ^1^
	Systolic blood pressure, mmHg	120	126	0.070
Physiology	Heart rate, bpm ^2^	92	84	0.004
	Temperature, °C	37.3	36.7	<0.001
	Total NEWS score	3.47	1.79	<0.001
Paraclinical	C-reactive protein, mg/L	144.1	50.5	<0.001
	Leucocytes, ×10^9^/L	10.2	10.6	0.688
	Neutrophils ×10^9^/L	8.67	8.48	0.839
Symptoms at admission, NRS	Average pain	4.12	4.09	0.959
	Worst pain	5.40	5.40	0.997
	Tiredness	5.60	5.80	0.626
	Drowsiness	5.65	5.32	0.430
	Nausea	1.98	2.71	0.115
	Appetite	4.62	4.98	0.515
	Shortness of breath	3.69	3.41	0.585
	Depression	3.38	3.64	0.611
	Anxiety	2.80	2.97	0.719
	Well-being	4.61	4.81	0.601
	Sleep	5.02	3.91	0.017
	Constipation	2.96	3.40	0.455

^1^ Analyzed by independent sample *t*-test. ^2^ beats per minute.

**Table 4:** Change from admission to discharge or 10 days.

	Difference ^1^	Infection Group, *n* = 62. Mean	Non-Infection Group, *n* = 157. Mean	*p*-Value ^2^
Physiology	Diff systolic blood pressure	−15.06	−2.56	0.001
	Diff heart rate	9.51	0.43	0.005
	Diff temperature	0.72	0.04	<0.001
	Diff total NEWS score	1.60	−0.05	<0.001
	Diff C-reactive protein	66.4	−0.7	<0.001
Paraclinical	Diff leucocytes	1.48	0.08	0.051
	Diff neutrophils	1.59	0.32	0.057
Symptoms	Diff average pain.	1.04	1.40	0.440
	Diff worst pain.	1.98	1.40	0.302
	Diff tiredness	1.26	1.45	0.667
	Diff drowsiness	1.36	1.17	0.670
	Diff nausea	0.57	0.92	0.409
	Diff appetite	0.82	1.37	0.309
	Diff shortness of breath	0.85	1.06	0.605
	Diff depression	0.49	0.59	0.793
	Diff anxiety	0.24	0.71	0.236
	Diff well-being	0.49	1.05	0.232
	Diff sleep	1.09	0.63	0.378
	Diff constipation	1.34	1.25	0.870

^1^ Difference is observations at admission—discharge/after 10 days. ^2^ Analyzed by the independent sample *t*-test.

## Data Availability

The corresponding author has full control of all primary data. The dataset generated and/or analyzed is available from the corresponding author on request.

## Appendix A

**Table A1:** Course of disease during stay infection group, *n* = 62.

		Admission Mean	Day 10. Mean	Difference	95% Confidence Interval of the Difference	*p*-Value, *
Physiology	Systolic blood pressure, mmHg	122	137	−15.06	−22.24–(−7.87)	<0.010
	Heart rate, bpm	90	80	9.51	4.58–14.44	<0.001
	Temperature, °C	37.3	36.5	0.72	0.39–10.58	<0.001
	Total NEWS score	2.96	1.36	1.60	0.87–2.34	<0.001
Paraclinical	C-reactive protein, mg/L	147.8	81.4	66.41	41.70–91.19	<0.001
	Leucocytes, ×10^9^/L	9.9	8.4	1.48	0.29–2.66	0.016
	Neutrophils, ×10^9^/L	8.36	6.77	1.59	0.43–2.74	0.008
Symptom, NRS	Average pain	4.22	3.17	1.04	0.28–1.81	0.008
	Worst pain	5.45	3.48	1.98	0.95–3.00	<0.001
	Tiredness	5.66	4.40	1.26	0.57–1.94	<0.001
	Drowsiness	5.81	4.45	1.36	0.63–2.09	<0.001
	Nausea	2.09	1.51	0.57	−0.13–1.28	0.109
	Appetite	4.58	3.76	0.82	−0.21–1.85	0.114
	Shortness of breath	3.57	2.72	0.85	0.27–1.43	0.005
	Depression	3.36	2.87	0.49	−0.08–1.05	0.087
	Anxiety	2.71	2.47	0.24	−0.44–0.93	0.475
	Well-being	4.51	4.02	0.49	−0.22–1.20	0.122
	Sleep	5.15	4.07	1.09	0.23–1.90	0.010
	Constipation	3.07	1.73	1.34	0.35–2.33	0.009

* two-sided, paired sample *t*-test.

**Table A2:** Course of disease during stay, non-infection group, *n* = 157.

		Admission Mean	Day 10. Mean	Difference	95% Confidence Interval of the Difference	*p*-Value *
Physiology	Systolic blood pressure, mmHg	126	129	−2.56	−6.38–1.25	0.186
	Heart rate, bpm	84	84	0.43	−2.99–3.86	0.803
	Temperature, °C	36.7	36.7	0.04	−0.11–0.20	0.593
	Total NEWS score	1.73	1.77	−0.45	−0.51–0.42	0.848
Paraclinical	C-reactive protein, mg/L	56.0	56.6	−0.69	−9.67–8.29	0.879
	Leucocytes, ×10^9^/L	10.7	10.6	0.08	−0.74–0.90	0.846
	Neutrophils, ×10^9^/L	8.73	8.41	0.32	−0.43–1.06	0.402
Symptom, NRS	Average pain	4.28	2.88	1.40	0.88–1.92	<0.001
	Worst pain	5.48	4.08	1.40	0.79–2.01	<0.001
	Tiredness	6.07	4.62	1.45	0.92–1.97	<0.001
	Drowsiness	5.55	4.38	1.12	0.65–1.69	<0.001
	Nausea	2.77	1.85	0.92	0.46–1.38	<0.001
	Appetite	4.83	3.47	1.37	0.81–1.92	<0.001
	Shortness of breath	3.48	2.41	1.06	0.56–1.57	<0.001
	Depression	3.71	3.12	0.59	0.11–1.08	0.017
	Anxiety	3.08	2.36	0.71	0.28–1.15	0.002
	Well-being	5.09	4.05	1.05	0.49–1.61	<0.001
	Sleep	3.74	3.12	0.63	0.02–1.23	0.042
	Constipation	3.61	2.36	1.25	0.61–1.89	<0.001

* Two-sided, paired sample *t*-test.

**Table A3:** Characteristics of integrated cancer care (*n* = 81) and palliative care cancer patients (*n* = 136). Two missing (information not available).

		Integrated Cancer Care *n* = 81.	Palliative Cancer Care *n* = 136.
Receiving antibiotics, *n* (%)		24 (30)	37 (27)
Radiology, *n* (%)	X-ray	26 (32)	52 (38)
	Computer tomography	34 (42)	47 (35)
	Ultrasound	7 (9)	15 (11)
	Magnetic resonance imaging	21 (26)	10 (7)
Interventions, *n* (%)	Radiological intervention ^1^	7 (9)	11 (8)
	Rehydration	38 (47)	68 (50)
	Nutrition	10 (12)	15 (11)
	Transfusion	10 (12)	23 (17)
	Surgery	1 (5)	0
In-hospital mortality, *n* (%)		4 (5)	26 (19)

^1^ e.g., drains and stenting.
